# Neuroaging through the Lens of the Resting State Networks

**DOI:** 10.1155/2018/5080981

**Published:** 2018-01-15

**Authors:** Filippo Cieri, Roberto Esposito

**Affiliations:** ^1^Forensic Mental Health Residential Rehabilitation “Quadrifoglio”, Rosello, Chieti, Italy; ^2^Department of Radiology, Azienda Ospedaliera Ospedali Riuniti Marche Nord, Pesaro, Italy

## Abstract

Resting state functional magnetic resonance imaging (rs-fMRI) allows studying spontaneous brain activity in absence of task, recording changes of Blood Oxygenation Level Dependent (BOLD) signal. rs-fMRI enables identification of brain networks also called Resting State Networks (RSNs) including the most studied Default Mode Network (DMN). The simplicity and speed of execution make rs-fMRI applicable in a variety of normal and pathological conditions. Since it does not require any task, rs-fMRI is particularly useful for protocols on patients, children, and elders, increasing participant's compliance and reducing intersubjective variability due to the task performance. rs-fMRI has shown high sensitivity in identification of RSNs modifications in several diseases also in absence of structural modifications. In this narrative review, we provide the state of the art of rs-fMRI studies about physiological and pathological aging processes. First, we introduce the background of resting state; then we review clinical findings provided by rs-fMRI in physiological aging, Mild Cognitive Impairment (MCI), Alzheimer Dementia (AD), and Late Life Depression (LLD). Finally, we suggest future directions in this field of research and its potential clinical applications.

## 1. Introduction 

 Daily life mental activity occurs often in the absence of external stimuli. During this state of consciousness, we are engaged in recording bodily sensations (somesthetic and vegetative); we experience the free association of thoughts that involves memory (past experience, inner dialogue, mental images, and emotions planning future events). In absence of external stimuli, mind jumps from one thought to another with fluidity and simplicity, what William James (1890) called “flow of consciousness.” This spontaneous brain activity was called Random Episodic Silent Thinking (REST; [1]), emphasizing the free and errant nature of this mode of thinking, partly in contrast with the engagement of mind during cognitive tasks.

Modern neuroimaging techniques, such as Positron Emission Tomography (PET), Functional Magnetic Resonance Imaging (fMRI), and Magnetoencephalography (MEG), have allowed studying brain* in vivo* showing the intersection of anatomy and functions. Particularly, fMRI is a relative noninvasive neuroimaging technique used for many years to study brain eloquent areas activation during task execution. In the last two decades fMRI was used to study low-frequency fluctuations of cerebral hemodynamics (0.01–0.1 Hz) that are associated with complex brain “activation maps” temporally correlated across the brain and correspond to functional RSNs [2]. RSNs are thought to represent the neuronal baseline activity of human brain in the absence of external stimuli and identify the presence of functionally distinct networks [3, 4]. This brain baseline activity represents a model of mind, enhanced when the focus of attention shifts from external to internal self-referential state [5]. Even if today some skepticism persists about the neural substrate of spontaneous brain activity, this theory is supported not only by rs-fMRI but also by observing electric activity, hemodynamic and metabolic parameters, spontaneous fluctuations of membrane potential, spontaneous spikes, and neurotransmitter release [6]. Despite being the object of a thriving field of clinical research, the investigation of aging through the lens of the RSNs is in its early days.

## 2. Default Mode Network and the Other Resting State Networks

**Figure 1:**
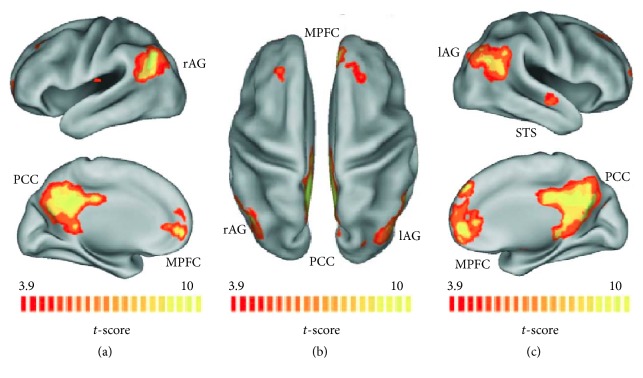
*Default Mode Network (DMN)*: maps of healthy subjects obtained by means of Independent Component Analysis (ICA) and superimposed on inflated Talairach template: (a) lateral and medial views of the left hemisphere. (b) Dorsal view. (c) Lateral and medial views of the right hemisphere. DMN areas are labeled (MPFC: medial prefrontal cortex; PCC: posterior cingulate cortex; lAG and rAG: left and right angular gyrus; STS: superior temporal sulcus). The figure is derived from the following study: [7].

The identification of the specific brain areas constituting RSNs dates back to early 21st century as a result of a group of scientists using PET imaging [10, 11]. The first cerebral areas identified constituted a network involving both hemispheres: medial prefrontal cortex (MPFC), the posterior cingulate cortex (PCC), the angular gyrus (AG), and hippocampal regions (HP) (Figure 1). The neural network was called DMN and it is engaged when mental activity is internally directed, for example, when a person is left “undisturbed” to think of himself, about his past or his future. DMN is also referred to as the task-negative network because these regions are typically deactivated during execution of attention demanding tasks [12, 13]. DMN supports mental processes characterized by future planning, autobiographical memory, moral dilemmas, records of bodily sensations, and self-referential mental activity [14], showing instead reduced activation when a person is engaged in a cognitive task. 

Within the DMN the MPFC supports self-referential mental processes, monitoring psychological states [15], and receives interior (bodily sensations, proprioceptive) and external inputs (visual, auditory) supporting their integration and processing. Ventral part of MPFC (VMPFC) shows different interconnections with the limbic system, particularly with amygdala, mediating visceromotor aspects connected to emotions [16], a preconscious process that becomes conscious through modulations of other brain regions. MPFC shows important neural connections with HP; in fact DMN supports memory through two subsystems: the temporal-mesial subsystem, associated with mnemonic processes and predominantly made up of HP, that shows high connectivity with PCC/Precuneus (PCC/PrC) and Inferior Parietal Lobule (IPL); the second subsystem connected to the dorsal part of MPFC (DMPFC) is activated during mental situations of self-exploration and sensations. The results suggest that self-referential mental activity engages preferential the MPFC subsystem [17]. Another important DMN region is the Anterior Cingulate Cortex (ACC), associated with the control of different functions such as selection or inhibition of responses, conflict monitoring, and errors detection. Although the precise functions of the DMN remain a matter of debate, mounting evidence points to two distinct DMN subsystems that may mediate dissociable aspects of internal mentation, namely, memory-based construction/simulation (medial temporal lobe subsystem) and introspection about mental states (DMPFC subsystem) [18, 19]. Stronger resting state functional connectivity within the DMN has been associated with higher frequencies of spontaneous thought and engagement in mind wandering [20]. Nevertheless, regions outside the DMN have also been implicated in mind wandering, in particular executive control systems, yet the exact role of these systems in mind wandering remains not clear [21].

**Figure 2:**
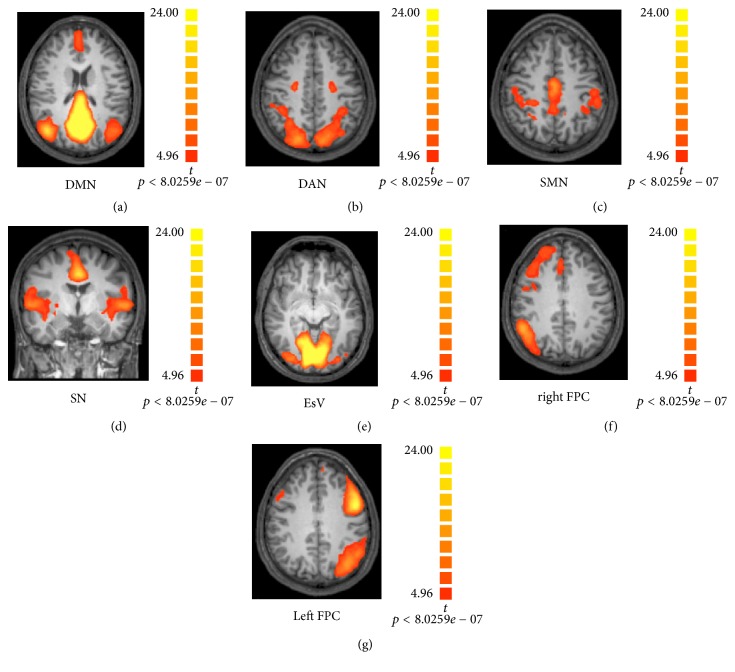
*Resting State Networks (RSNs)*: maps of healthy subjects obtained by means of Independent Component Analysis (ICA), overlaid on Talairach template (T1 weighted images), showed in radiological convention. DMN: Default Mode Network; DAN: Dorsal Attention Network; SMN: Sensory Motor Network; SN: Salience Network; EsV: Extrastriate Visual; FPC: Frontoparietal Control Network. The figure is derived from the following study: [8].

DMN represent only one network, because to date, others RSNs have been identified [3, 22, 23]: the Salience Network (SN); the Frontoparietal Network (lateralized in both hemispheres; FPN); the primary Sensory Motor Network (SMN); the Extrastriate Visual System (EsV); and the Dorsal Attention Network (DAN). RSNs are linked by anatomical connections and engaged in complex patterns of neuronal communication and signaling (Figure 2).

The DAN, which includes Inferior Parietal Sulcus (IPS), Frontal Eye Field (FEF), ACC, and bilateral Middle Temporal Gyrus (MidTempG), has received much attention. It is also called task-positive network because, as we mentioned above, its main regions are commonly activated in tasks demanding attention and mental control [24, 25]. DMN and DAN show a pattern of anticorrelated activity in both task and resting state studies [26] and their competitive relationship may represent a cerebral mechanism supporting cognitive functions [27, 28], switching focus between internal (supported by DMN) and external channels (supported by DAN) [9], when the system works properly.

Recently, explaining the complex communication between different RSNs has been proposed by a “triple network” hypothesis [29]. This hypothesis involves three networks: central executive network (CEN), SN, and DMN [29, 30]. CEN and SN increase activity during cognitive or affective processes while the DMN shows decreased activation during tasks in which self-referential activity is not involved [31]. Interestingly modifications of interconnections among DMN, CEN, and SN were observed in many psychiatric and neurological disorders, for instance, Attention Deficit Hyperactivity Disorder (ADHD; [32]), psychosis [33], and depression [34], and their functional or dysfunctional activity is crucial during healthy or pathological aging, such as MCI [35] and AD [36].

## 3. Healthy and Pathological Aging

Many elders, over 65 years old, live in happy and healthy life conditions. Some changes in memory functions are considered a normal part of the aging process, but almost 40 per cent of older adults experience during their life a mild cognitive decline often with an impairment of memory. This impairment may occur in different areas of memory such as visual or verbal memory, visuospatial abilities, immediate memory, or the ability to name objects. When there is no underlying medical condition causing this memory loss, it is known as age-associated memory impairment which is considered a part of the normal aging process. These preclinical conditions differ from neurodegenerative diseases like AD that are characterized by specific biomarkers (neuropsychological, cognitive, biological, biochemical, and neurological).

AD deeply affects healthy citizen as well as the wealth of public health systems and, with the growing rate of elderly people in western countries, is becoming a health/economic issue of epidemic proportions [38]. A recent intriguing and promising approach in the field of dementia concerns the use of advanced neuroimaging techniques in association with neuropsychological evaluation and quantification of biological markers to identify subjects with high risk of developing AD in preclinical condition (i.e., Apolipoprotein E4-APOE 4 carriers) [39, 40]. Indeed, advanced neuroimaging techniques may provide a valid tool to differentiate brain changes due to physiological aging [41, 42] and abnormal changes possibly underlying neurodegenerative pathologies such as AD [43–45].

Several studies explored modification of DMN connectivity in healthy elders, MCI patients, and AD [46, 47] in order to find useful biomarkers for early diagnosis and start of targeted treatments. DMN functional connectivity impairment correlates with cognitive decline, impairment of executive functions, and working memory [48]. We have strong evidences that allow us to identify specific brain signs prior to the clinical phase of AD [9, 49]. In fact, preclinical AD has been associated with early detection of pathological modifications involving different cerebral regions: reduced DMN connectivity in normal aging and in preclinical stages of dementia [46, 47], especially between anterior and posterior DMN components (MPFC and PCC), was observed. Functional connectivity declines with age mainly affecting DMN; however there are some evidences of increased connectivity with age interpreted as compensatory mechanisms during aging process [50]. Indeed, results observed are controversial, with some studies showing decreased connectivity and other showing opposite results [51], influenced by the data analysis approach used. For example, the seed based method of analysis showed very controversial results with decreased connectivity between HP and PCC or increased connectivity between Prefrontal Cortex (PFC) and HP and between PFC and PCC [52] and the increased connectivity in PFC was interpreted as a compensation mechanism during aging processes. Another interesting approach for rs-fMRI data analysis is the independent component analysis (ICA). The results often show decreased connectivity within DMN, also during tasks, worsening with the onset of AD [53]. In some studies, the reduction of connectivity of DMN is associated with increased connectivity in other networks such as FPN [54]. The most controversial result is represented by the interpretation of the result observed as prodromal brain changes of neurodegenerative diseases or compensatory mechanisms to counteract cognitive decline [3, 43].

As regards fMRI modifications observed in AD, several studies highlighted the importance of amyloid deposition in specific regions of DMN resulting in decreased functional connectivity. The vulnerability of these regions is unknown; however, it correlated with multiple predisposing factors (vascular damage, iron deposition, and inflammatory changes). The PCC/PrC node of DMN received great attention showing significant modification in normal aging and moreover in AD, indicating amyloid deposition and reduction of metabolism, especially in APOE E4 patients [49, 55]. PCC/PrC supports self-referential mental activity, memory functions, but also attention, regulating the internal and external focus of thoughts. It has strong functional and anatomical connections with other brain regions, such as the HP, VMPFC, and ACC [55]. The failure of deactivation during task performance [56] and reduction of functional connectivity [57] of PCC may represent an early AD biomarker. Moreover, PCC is constituted of different functional subparts each one exchanging information with the other RSNs [58]. Rearrangement of DMN intrinsic connectivity especially within PCC and left-IPL may represent a compensatory mechanism to counteract neuronal dysfunction in MCI patients and even more in those MCI patients that during follow-up converted in AD [43, 47, 59]. As a confounding factor, DMN modifications observed in AD are common findings in many other mental disorders [60]. Crucially, the extent to which a concurrent task demands attention, and the meta-awareness required to self-identify and self-classify thoughts, restricts its broader application to clinical populations. Evidence suggests that, in the context of low task demands subjects report more off-task thoughts, compared to reports during more demanding tasks [61]. To date, there is a lack of available paradigms to investigate mind wandering in a context free from external additional loads on attention and working memory processes. Also for these reasons mind wandering assessment is particularly difficult in neurodegenerative diseases or psychiatric conditions, where the integrity of the DMN is significantly compromised [60, 62, 63]. Significant intratemporal lobe connectivity, coupled with relatively weaker connectivity of temporal lobe regions to PCC and DMPFC within DMN, is associated with mind wandering [64]. The observed mind wandering frequency is strongly linked to relatively circumscribed connectivity within lateral temporal lobe regions and concomitant decreased connectivity between temporal regions and midline cerebral regions; these results are in contrast with other studies showing increased connectivity within and between a distributed range of DMN nodes during mind wandering [20, 65].

**Figure 3:**
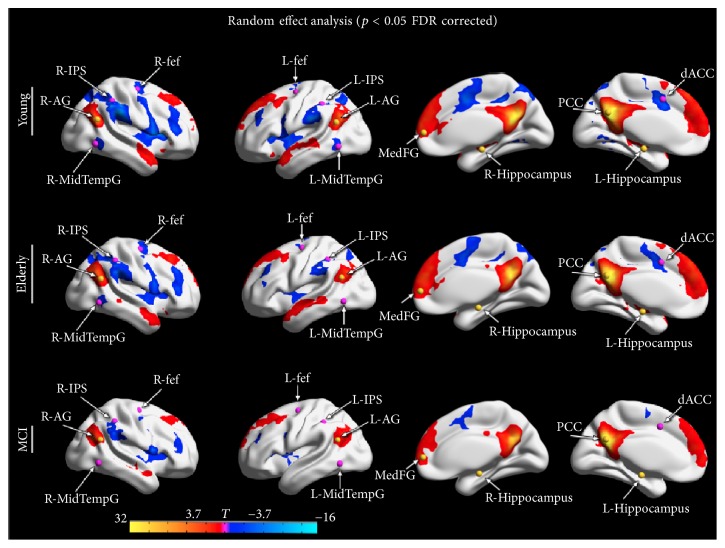
*Default Mode Network (DMN) and Dorsal Attention Network (DAN) anticorrelations*: Seed based connectivity maps obtained from random effects group analyses, superimposed on inflated Talairach template. The maps show DMN-DAN anticorrelations for healthy young subjects, healthy elders, and Mild Cognitive Impairment (MCI) patients. The figure is derived from the following study: [9].

An interesting hypothesis that sheds light on aging processes as AD biomarkers is the modification of DMN-DAN anticorrelation pattern [9]. A decreased DMN-DAN anticorrelation seems to be part of the normal aging process and probably its impairment explains partially cognitive decline in MCI and AD patients. In fact, brain is intrinsically organized into anticorrelated networks [26], supporting behaviour and cognitive functions [27, 28] and representing a brain physiological function starting up when the focus of attention switches between self-referential mental activity and external attention [66]. Interestingly this DMN-DAN negative correlation modifies its function during life span: it appears during the first year and strengthens during the second year of life [67], becoming more robust in adults to support the development of executive functions and working memory [68]. Finally decreased DMN-DAN anticorrelation characterizes healthy elders [69] and may represent neuronal substrates of initial cognitive decline (Figure 3).

As concerns the triple network model, altered within-network connectivity among DMN, CEN, and SN was observed in healthy subjects at risk of AD (APOE4 carriers) [33]. The preserved connectivity within triple network system may explain preserved cognition in the cognitively normal APOE4 allele carriers. Moreover, the normal functioning of triple network interaction may positively affect the compensation of initial cognitive decline as observed in a recent study showing reduced connectivity within DMN between PCC and HP and from thalamus to PCC and increased connectivity within SN from dorsal ACC to striatum, from the CEN to the DMN, and from the SN to CEN in MCI patients compared with AD patients and healthy controls [67].

Finally, interesting results arise from the intersection of imaging data and molecular data in normal aging and AD tracing by means of PET amyloid a*β*1–42 (Pittsburgh Compound B (PiB)-PET) and Tau neurofibrillary tangles (AV1451-PET). Indeed, individuals with preclinical AD have relied on associations with in vivo measures of amyloid pathology. While many studies have reported decreased functional connectivity with increased amyloid (a*β*) burden in the medial temporal lobe, posterior midline, and parietal regions [38], other studies have reported regions of both increased and decreased connectivity with elevated amyloid [70]. With the recent advent of in vivo Tau-PET tracers it is now possible to extend investigations on fcMRI in a sample of cognitively normal elderly humans to regional measures of Tau. In a recent study [71], the authors showed that amyloid-positive (a*β*+) individuals were characterized by increased connectivity in DMN and SN when neocortical Tau levels are low, whereas a*β*+ individuals demonstrate decreased connectivity in these networks as a function of elevated Tau-PET signal. This pattern suggests a hyperconnectivity phase followed by a hypoconnectivity phase in the course of preclinical AD [71].

## 4. Late Life Depression

Another important condition that affects people after 60–65 years of age, with emotional and cognitive impairment, is Late Life Depression (LLD), a common mental disability in elderly population characterized by the presence of depressed mood, loss of interest or pleasure in daily activities, sleep disturbance, appetite disturbance, and cognitive and somatic symptoms. This specific mood disorder tends to increase considering the progressive aging population. Its prevalence rates can range from 1% to 4% for major and up to 13% for minor depression [72]. FMRI studies have shown varied and controversial results reporting modifications of cerebral connectivity in elders with depression [73, 74], but there is a strong association between depressive symptoms and MCI among older adults, although the neural correlates of this relationship are not fully understood. We surely agree that patients with LLD show often signs of cognitive decline and patients with MCI can complain about anxiety or depression symptoms; most importantly, both show higher conversion rate to AD [75].

**Figure 4:**
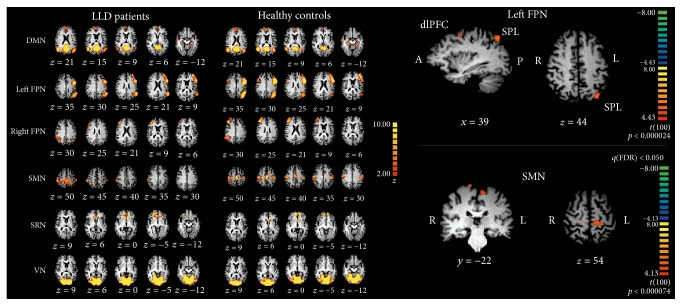
*Resting State Networks (RSNs) in Late Life Depression (LLD)*:* left panel* illustrates Default Mode Network (DMN), Somatomotor Network (SMN), left and right Frontoparietal Network (FPN), Self-Referential Network (SRN), and Visual Network (VN), overlaid on Talairach template (T1 weighted images), showed in radiological convention. Left: LLD patients; Right: healthy controls.* Right panel* illustrates between-group differences. Top: Left FPN; Down: SMN. Contrast maps are overimposed on Talairach template (T1 weighted images) in radiological convention. The figures are derived from the following study: [37].

One of most important signs of these patients is the lack of DMN suppression during cognitive tasks [76], closely linked to the enhanced ruminative processes. Indeed, these patients demonstrate more engagement in mentalizing about the self and past autobiographical experiences, focusing on negative thoughts. The DMN area most involved in depressed patients is the MPFC, including the ventromedial and dorsolateral part. As we have mentioned above MPFC supports self-referential mental processes, monitoring psychological states [15]; it is critical to social cognition, self-reference, emotional decision-making, and emotion regulation. This region has been implicated not only in LLD, but more in general in the development of anxiety and depression disorders [77, 78]. The functions of this area are tied to its structural and functional connections to numerous key areas of DMN such as the PCC/PrC and HP, key nodes of memory retrieval, as well as the amygdala and insula, key region of the SN, caudate and putamen, core of the reward system, superior temporal sulcus (STS) and middle temporal cortex, and central nodes of theory of mind [79]. The involvement of all these structures and functions explains the extended presence of such complex and disabling affective, cognitive, and somatic symptomatology. It is not fully understood whether the DMPFC is more an “affective” region and the VMPFC is more an “emotion regulation” region or vice versa, but Aghajani and colleagues [80], for example, propose the DMPFC as an emotional assessment/processing region and the VMPFC with functions of emotions regulation. The increased connectivity shown by these patients between specific nodes of DMN, as the subgenual part of ACC (sgACC) and the PCC, could be detrimental to cognitive processes [60, 81]. However, connectivity between the SN (i.e., anterior insula, dorsal ACC) and the anterior nodes of DMN [82] as well as between the insula and the amygdala [83] seems enhanced in depressed individuals when compared with controls and these results are coherent with the more engagement in mentalizing about the self and past autobiographical experiences and focusing on negative thoughts demonstrated in these patients. In our recent study [37] we showed a significant modification in the left FPN (lFPN) characterized by increased connectivity in the left Superior Parietal Lobule (SPL) and left DLPFC in LLD group (Figure 4). The lFPN is implicated in language and working memory processing [84, 85] and the significant modification found in our study could justify some typical cognitive symptoms in LLD, assuming an inability to regulate the activation of an important area for specific cognitive tasks, as the case of working memory, impaired in LLD, MCI, and AD. Alterations in SPL connectivity are less often described in the literature to be associated with LLD [86], while the structural connectivity using Diffusion Tensor Imaging (DTI) between SPL of both cerebral hemispheres seems to discriminate patients with depression from healthy controls [87]. Parietal cortex provides a coherent self-representation across space and time [88] and is implicated in top-down control of attention, with sensorimotor integration, balancing internally and externally directed attention [58]. In a recent comparative study between schizophrenic patients and major depressive disorder, the authors found significant reduction in connectivity within the posterior nodes of DMN and SPL in patients with depression hypothesizing an imbalance between internally and externally directed attention and mental state attribution [89]. In fact, SPL appears to be involved in disengaging or maintaining attention to visual and tactile stimuli [90, 91], as demonstrated in patients with tumor localized in left SPL, suggesting that SPL is important in maintaining internal representation of body states [92]. This region continues onto the medial surface of the hemisphere as the PrC, forming rectangular-shaped area involved in mental imagery and recall of personal experiences; thus, like the MPFC, it is part of the DMN of the brain, engaged during activities such as daydreaming and introspection [93], both impaired in depression disorders. Also, epileptic seizures in the SPL cause disturbances of the body image; thus it is also possible to hypothesize an increased attention for the internal representation of body states in patients with LLD.

## 5. Conclusion

For many years, modern neuroimaging techniques neglected brain spontaneous activity, focusing only on changes evoked by external tasks. Within the past two decades, rs-fMRI has taken off as a major tool to study brain in vivo, especially for those patients less cooperative, offering detailed and clear information about the spontaneous brain dynamics in both physiological and pathological conditions. Indeed, the common target of the neuroscientists is to identify early biomarkers before clinical outbreak of AD and advancements in genetics, neurobiology, and neuroimaging techniques allowed researchers to hypothesize the mechanisms underlying these disorders.

Abnormal functional connectivity has been reported in several neurologic diseases even if these results remain controversial. In the first rs-fMRI studies the results showed intranetwork connectivity modifications while more recent studies underlie altered interactions between different RSNs. In recent years, neurological disorders have better understood in its specific neural correlates and some shared results have been achieved, as, for example, the reduced DMN connectivity in normal aging and in preclinical stages of dementia [46, 47], especially between anterior and posterior DMN components. Moreover, the posterior nodes of DMN have received great attention showing significant modification in normal aging and moreover in AD, indicating amyloid deposition and reduction of metabolism, also in preclinical conditions as observed in APOE E4 patients [49, 51]. Other investigations in this area have demonstrated that PCC/PrC supports self-referential mental activity, memory functions, but also attention in regulation of internal and external focus of thoughts. Thus, the lack of this deactivation during task performance [56] and the reduction of functional connectivity [57] of PCC may represent an early biomarker of AD.

Another important chapter within the understanding of healthy and pathological aging has been the anticorrelation of task-negative and task-positive networks. In fact, decreased DMN-DAN anticorrelation has been observed in a recent study comparing young subjects, healthy elders, and MCI patients [69] hypothesizing that reduced anticorrelations may be considered a neuronal substrate of aging brain supporting cognitive decline. As concerns the triple network model, healthy subjects at risk of AD (APOE4 carriers) have shown altered within-network connectivity among DMN, CEN and SN [33]. The preserved connectivity within triple network system may explain preserved cognition in the cognitively normal APOE4 individuals.

Finally, another disease recently investigated through the rs-fMRI in aging is LLD, with important emotional and cognitive impairment. One of the most important areas involved in the LLD is the MPFC, for its implication in typical mental activity in depressed patients, such as self-reference, social cognition, emotional decision-making, and emotion regulation. LLD patients are more engaged in metalizing about self and past autobiographical experiences and focusing on negative thoughts. These individuals are characterized by inability to suppress DMN activity during cognitive task, coherent with these enhanced overthinking processes. Connectivity between SN and the anterior nodes of DMN as well as between the insula and the amygdala is a second feature coherent with their tendency to ruminate. Moreover, reduction in connectivity within the posterior nodes of DMN and SPL was observed in depressed patients, coherent with an imbalance between internally and externally directed attention and mental state attribution [89]. This imbalance seems one of the causes of cognitive impairment as a consequence of inability to focus on a specific cognitive task, because the attentive resources are disengaged and hijacked on emotional aspects, driving the ruminative elaborations and this is crucial and detrimental to cognitive processes.

Even if rs-fMRI represents a promising tool to study brain there are many confounding factors to be clarified [94]. For example, there is no control system to check if patients fall asleep during MRI scans; moreover, the great variety of methods of MRI data analysis contributes to create an “excess” of heterogeneity of results. Finally, fMRI records brain modifications of neurovascular coupling BOLD signal. Several diseases modify this signal with a consequent nonquantifiable effect on BOLD signal. Future research will take benefit by the combination of different methods such as MEG, Electroencephalography (EEG) and cerebral perfusion, and molecular data.

Functional connectivity has contributed substantially to the field of Alzheimer's research showing cerebral modifications even before symptoms arise and is emerging as a promising biomarker for longitudinal studies.
